# Efficient Parahydrogen-Induced ^13^C Hyperpolarization on a Microfluidic Device

**DOI:** 10.1021/jacs.4c03271

**Published:** 2024-06-25

**Authors:** Sylwia J. Barker, Laurynas Dagys, Malcolm H. Levitt, Marcel Utz

**Affiliations:** † School of Chemistry, University of Southampton, Southampton SO17 1BJ, United Kingdom; ‡ Institute of Microstructure Technology, Karlsruhe Institute of Technology, Karlsruhe 76131, Germany; ¶ Institute of Chemical Physics, Vilnius University, Vilnius 01513, Lithuania

## Abstract

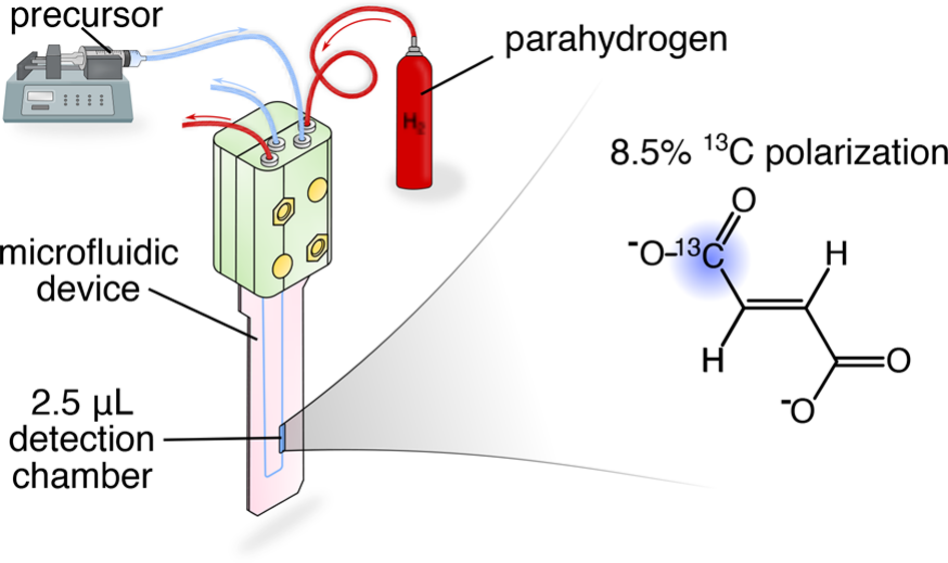

We show the direct production and detection of ^13^C-hyperpolarized fumarate by parahydrogen-induced polarization (PHIP) in a microfluidic lab-on-a-chip (LoC) device and achieve 8.5% ^13^C polarization. This is the first demonstration of ^13^C-hyperpolarization of a metabolite by PHIP in a microfluidic device. LoC technology allows the culture of mammalian cells in a highly controlled environment, providing an important tool for the life sciences. In-situ preparation of hyperpolarized metabolites greatly enhances the ability to quantify metabolic processes in such systems by microfluidic NMR. PHIP of ^1^H nuclei has been successfully implemented in microfluidic systems, with mass sensitivities in the range of pmol/s. However, metabolic NMR requires high-yield production of hyperpolarized metabolites with longer spin life times than is possible with ^1^H. This can be achieved by transfer of the polarization onto ^13^C nuclei, which exhibit much longer *T*_1_ relaxation times. We report an improved microfluidic PHIP device, optimized using a finite element model, that enables the direct and efficient production of ^13^C-hyperpolarized fumarate.

## Introduction

Lab-on-a-chip (LoC) systems that can culture cells, cell aggregates, or tissues, are increasingly adopted as a research tool in the life sciences, especially in drug development.^1−3^ While this is partly driven by the widely recognized need to reduce animal testing, LoC cultures allow the use of human cells and can therefore provide more relevant models of human disease. Microfluidic technology enables precise control over the cellular growth environment, and offers high throughput and a high degree of reproducibility. In this way, cellular processes and functions as well as their response to external stimuli such as drugs,^4^ therapeutic targets,^5−7^ toxins,^8,9^ and oxygen or nutrient supply^10,11^ can be studied systematically. Microfluidic NMR^12−14^ allows noninvasive and real-time *operando* quantitative characterization of metabolic^15−17^ and chemical^18^ processes in LoC devices. However, sensitivity is limited in these systems due to their small size. Hyperpolarization of the nuclear spins^19^ could address this, but requires preparation of hyperpolarized species that can be metabolized by the cultured cells, with a lifetime of the spin order long enough to detect downstream metabolic products.

Hyperpolarized metabolites have great potential as contrast agents for magnetic resonance imaging (MRI) and magnetic resonance spectroscopic imaging (MRSI), providing real-time and quantitative information on active metabolic pathways in healthy and diseased tissues.^20,21^ This approach has been used *in vivo* for metabolic profiling of tumors such glioma,^22,23^ hepatocellular carcinoma, lymphoma,^24,25^ pancreatic^26^ and breast cancers.^27,28^ In this modality, relatively large amounts (several g) of hyperpolarized material (most commonly pyruvate) are prepared and injected intravenously into the patient. Preparation relies on either dissolution dynamic nuclear polarization^29,30^ or on low-field polarization transfer based on parahydrogen-induced polarization (PHIP).^31,32^ The batch mode of operation of these methods does not lend itself to LoC culture devices, where a steady supply of much smaller amounts of hyperpolarized metabolites is needed. In this case, preparation methods that operate continuously at flow rates compatible with microfluidic systems (up to a few μL/min) are required.^33^ Additionally, as the lifetime of hyperpolarized species is limited by nuclear relaxation, it is crucial to produce them directly on the microfluidic device, in immediate proximity of their usage.

PHIP makes it possible to enhance NMR signals by up to 5 orders of magnitude.^34,35^ It utilizes *para*-hydrogen (*p*-H_2_), the singlet nuclear spin isomer of molecular hydrogen, as a source of spin order. The nuclear spin order is transferred to a target molecule via a chemical reaction of *p*-H_2_ with an unsaturated molecule in the presence of an organometallic catalyst. The chemical reaction is followed by spin manipulations to transfer the parahydrogen-derived spin order to a desired nucleus, and may include purification steps to remove unwanted compounds.^36^

LoC devices can be used to implement some or all of these processes. Eills et al. have reported mass sensitives of the order of pmol/s for ^1^H in a microfluidic PHIP device^37^ based on diffusion of *p*-H_2_ through a silicone membrane, using propargyl acetate in methanol as a substrate. Barker et al. have subsequently shown that the same design can be used to directly hydrogenate acetylene dicarboxylic acid to produce ^1^H-hyperpolarized fumarate.^38^ However, the yield obtained in both cases falls short of the requirements for biological applications, particularly since further transformations such as purification and cleavage are required to effectively utilize the hyperpolarized material. To understand the interplay of the chemical, spatial and spin dynamics occurring on the microfluidic device proposed by Eills et al. Ostrowska et al.^39^ developed a finite element model of reaction and found that insufficient uptake of hydrogen was the limiting factor of the reaction.

In the present contribution, we report an improved device design, optimized using this finite element model to maximize hydrogen uptake. Additionally, we introduce a variable temperature control to regulate the temperature at the sample detection chamber. It is shown that these improvements, taken together, increase the yield to such a point that the production and detection of ^13^C-hyperpolarized fumarate becomes possible. To the best of our knowledge, this is the first report of PHIP-based ^13^C hyperpolarization in a microfluidic system.

## Materials and Methods

### Microfluidic Setup

The microfluidic device was manufactured from polycarbonate (PC) (Self Adhesive Supplies, UK) following the protocol given in ref (38). Briefly, devices were cut out with a LS3040 CO_2_ laser cutter (HPC Laser, United Kingdom) from three layers of polycarbonate sheet material with 0.25, 0.5, and 0.25 mm thickness for the top, middle, and bottom layers, respectively. The sample detection chamber in the middle layer and channels in the top layer were cut through, while the channels in the bottom and middle layers were engraved. After plasma activating using Corona Treater (Electro-Technic Products, USA), each layer was coated with 18 μL of plasticizer (5 v/v% dibutyl phtalate in isopropyl alcohol). Then the layers were dried for 15 min at 65 °C, assembled and bonded together under pressure and heat (5 tonnes, 85 °C).

**Figure 1:**
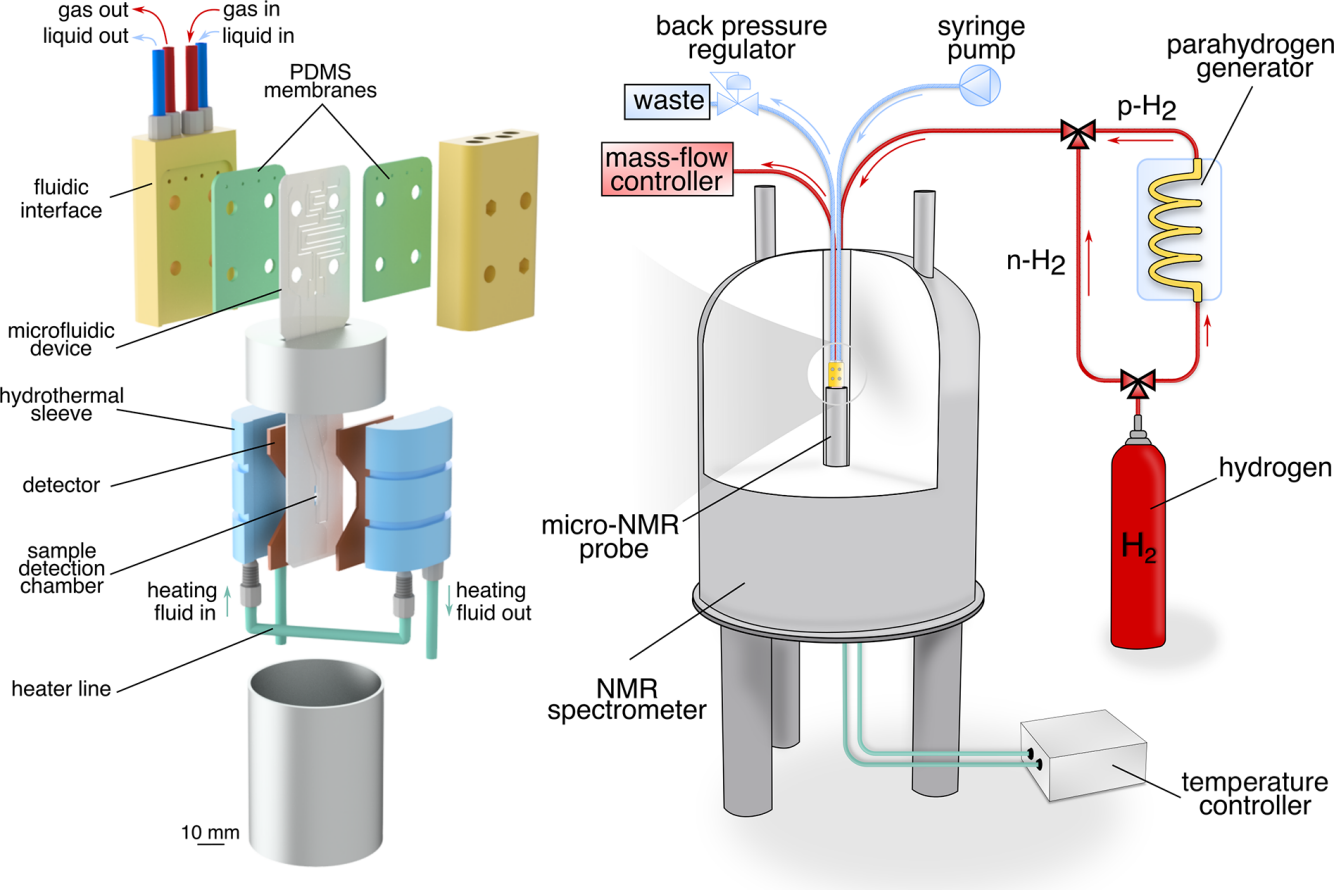
Experimental setup. The microfluidic chip assembly consists of a microfluidic device interposed between two PDMS membranes. These are held together by the fluidic interface that enables delivery of substrates into the chip. All experiments were performed inside of a high-field NMR spectrometer. Hydrogen/parahydrogen gas was supplied from a gas cylinder, while the precursor solution was introduced into the device using a syringe pump located outside of the spectrometer. The device was placed into the micro-NMR probe for detection. The probe was also equipped with hydrothermal sleeves regulated by a temperature controller that enabled efficient heating of the sample detection chamber.

The microfluidic assembly consisted of the chip interposed between two 1 mm PDMS membranes (Shielding Solutions, UK) held together by a fluidic interface (ProtoLabs, UK). Connectors for 1/16” fluid and gas lines (Cole Parmer, UK) facilitated the delivery of substrates onto the chip shown in Figure 1. PDMS membranes that covered the upper part of the chip served a dual purpose. First they promoted diffusion of hydrogen into the liquid channel and second, they enabled sealing of the assembly.

All experiments were conducted on a Bruker AVANCE III spectrometer operating at 11.7 T magnetic field. The microfluidic assembly was placed inside of a stripline-based micro-NMR probe for detection^14^ as shown in Figure 1. The probe was equipped with hydrothermal sleeves that housed a thermistor regulated by temperature controller, allowing efficient heating of the sample detection chamber only. The calibration of the heater was recorded by Rogers et al. and shows temperature fluctuations of less than 0.1 °C.^16^

The precursor solution was delivered into the chip using a syringe pump (Cole-Parmer, United Kingdom) located outside of the NMR spectrometer as illustrated in Figure 1. Hydrogen gas (gas purity 99.995%) was delivered from a cyliner located outside of the spectrometer at a flow rate set to 20 mL min^–1^ controlled using a mass-flow controller at the end of the gas line. The gas line was equipped with a valve selecting a flow of either hydrogen in thermal equilibrium or parahydrogen. Parahydrogen gas was obtained with 50% enrichment using a home-built parahydrogen generator filled with iron(III) oxide and cooled to 77 K.

All chemicals were purchased from Merck KGaA (Germany) and were used as received.

### Quantification of Hydrogen Uptake into the β-Chip

The uptake of hydrogen into the β-chip was quantified by flowing a solution of 20 mM sodium acetate dissolved in methanol-d_4_. In the gas channel, hydrogen in thermal equilibrium was supplied at 5 bar. The flow rate of hydrogen was controlled using a mass-flow controller positioned at the end of the gas line, set to a constant rate of 20 mL min^–1^. The flow rate of the liquid was varied from 2 to 20 μL min^–1^ in steps of 2 μL min^–1^  and the solution was left to equilibrate for 10 min at each flow rate. Then, 64 scans were acquired after the application of a  pulse with a recycle delay of 20 s. The NMR signal at 4.55 ppm was integrated to determine the H_2_ concentration.

### Finite Element Modeling

Finite element simulations were performed using COMSOL Multiphysics version 5.4. Figure 3a and Figure 3b show simulation domains for the α- and β-chips, respectively. The key functional components are the fluid channel, the sample chamber and PDMS membranes. The total volume of the β-chip was calculated as 7 μL. The simulation protocol and detailed results are given in the SI.

### Formation of ^13^C-Hyperpolarized Fumarate

The precursor solution contained 100 mM acetylene dicarboxylic acid [1-^13^C] disodium salt, 6 mM  catalyst and 200 mM sodium sulfite dissolved in D_2_O at 50 °C. The heater temperature was set to 58 °C. Flow rates from 2 to 16 μL min^–1^ in steps of 2 μL min^–1^ were studied. Parahydrogen pressure was set to 6 bar. The probe delivered nutation frequencies for ^13^C RF pulses of 12.5 kHz. Spectra were collected with a 200 ppm spectral width, and 8 k data points were acquired. Proton singlet order in [1-^13^C]fumarate was converted into the observable carbon magnetization using the singlet-to-heteronuclear-magnetization (S2hM) pulse sequence.^41^ The maximum efficiency was achieved using the following parameters: τ = 15.7 ms, *n*_2_ = 7, *n*_1_ = 7. The repetition delay was set to 60 s. The yield of fumarate was determined by comparing the integral of the fumarate peak at 6.8 ppm to the catalyst Cp* peak at 2.35 ppm (spectrum shown in the SI) and accounting for the difference in the number of protons. To calculate the enhancement factor for carbon polarization, the SNR of in the hyperpolarized spectrum was compared with the SNR obtained form a spectrum of 1 M d-glucose-1-^13^C averaged over 32 scans.

## Results and Discussion

**Figure 2:**
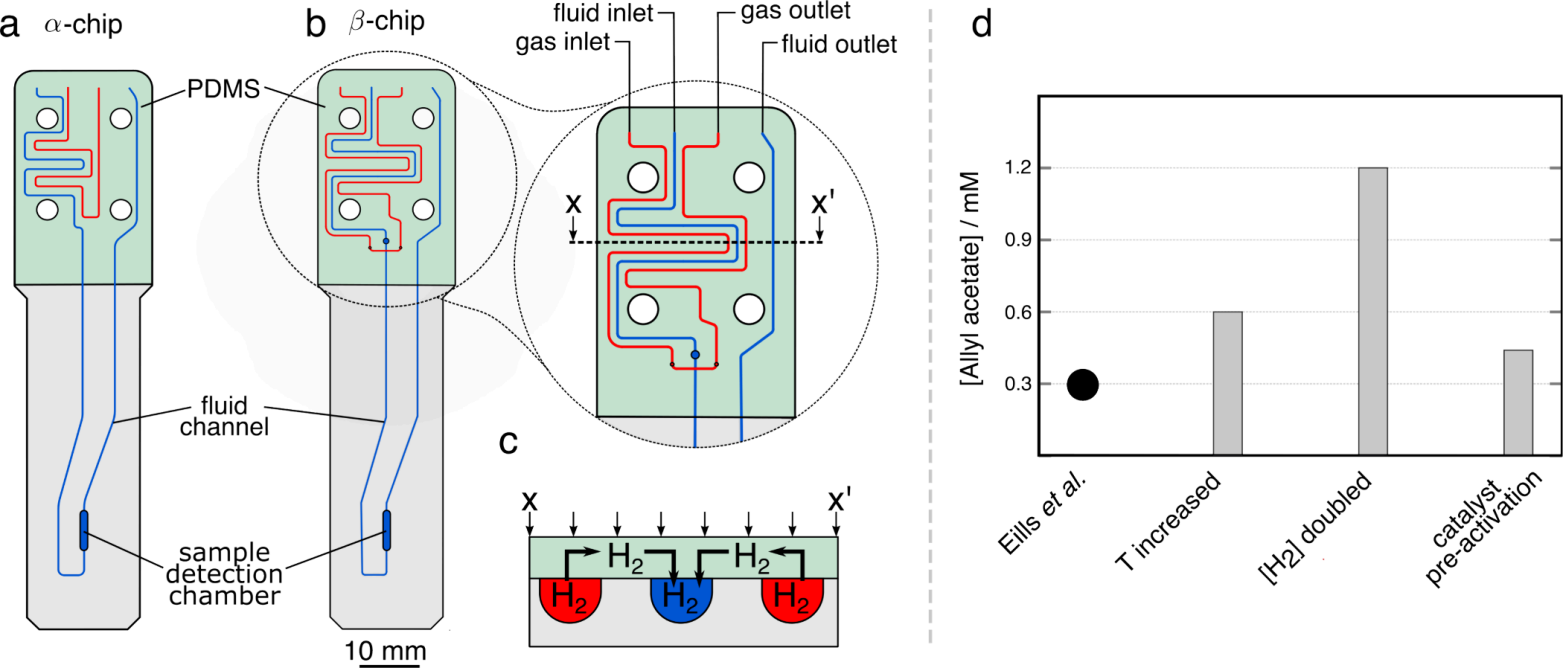
Top view of the microfluidic devices. (a) The α-chip used by Eills et al.^37^ Adapted from ref (39). Available under CC BY 4.0. Copyright Ostrowska et al. (b) The β-chip. The key functional area of the β-chip was enlarged. (c) Cross section of the β-chip. The PDMS membrane (green) acts as a bridge between the fluid (blue) and two gas (red) channels, enabling hydrogen to diffuse into the solution. (d) Concentration of allyl acetate reported by Eills et al.^37^ and three independent scenarios predicted by the model developed in ref (39).

The basic principle of operation of our PHIP device is shown schematically in Figure 2. The solution containing an unsaturated precursor flows through the channel indicated in blue, next to a channel containing parahydrogen gas under pressure, shown in red. Both channels are covered by a PDMS membrane, through which the molecular hydrogen diffuses efficiently (Figure 2c). Figure 2 compares the original chip design used by Eills et al.^37^ (a) with an improved design used here (b). The length of the fluid path has been increased, and the fluid path is now flanked by the hydrogen gas channel on either side.

An FEM model of the transport and chemical kinetics of the para-hydrogenation of propargyl acetate to allyl acetate has been previously reported.^39^Figure 2d shows the experimental yield of hyperpolarized allyl acetate reported by Eills et al.^37^ along with the prediction of the FEM model, whose kinetic parameters have been obtained from independent experiments at large scale.^39^ The model was used to explore three different hypothetical scenarios. In the first scenario all reaction rate constants were increased by a factor of 2, approximating a temperature increase by about 10 °C. As shown in Figure 2d, this leads to an increase in the yield by about a factor of 2 as expected. In the second case the partial pressure of hydrogen in the gas supply was doubled. The model predicts a massive increase in yield by a factor of 4. Finally, the catalyst activation rate was increased 10 times, simulating a situation where the protection group of the catalyst was replaced with one that is easier to remove. This led only to a modest increase in the yield. From these findings, we concluded that improvement of the hydrogen uptake was the most efficient way of increasing the yield of hyperpolarized product.

### Enhancing Hydrogen Uptake

**Figure 3:**
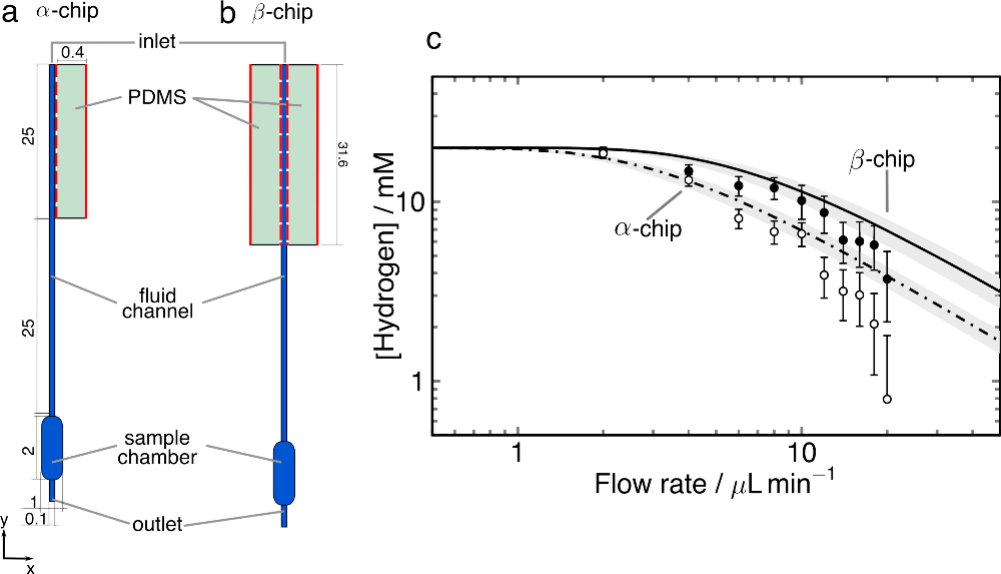
(a) α-Chip simulation domain. Adapted from ref (39). Available under CC BY 4.0. Copyright Ostrowska et al. (b) β-Chip simulation domain. (c) Hydrogen uptake into the chip as a function of flow rate of the liquid. The solid empty and black circles represent the NMR data for α- and β-chips, respectively. The solid and dash–dotted lines are the results of FEM simulations. The gray shadows represent ±1.5 μL error in the volume of the chip. Data for the α-chip was obtained from Eills et al.^37^

Experiments by Eills et al. had been carried out with hydrogen gas at 5 bar. Simply elevating hydrogen pressure in the chip is not viable as it tends to cause delamination and leakages, and high hydrogen pressures pose a safety hazard. Instead, the channel network can be modified to maximize the gas uptake. The fluidic design in the α-chip used by Ellis et al. consisted of one gas and one fluid channel in a side-by-side arrangement, with a PDMS membrane covering both channels and serving as a diffusion conduit for H_2_. The fluid channel in the β-chip design was positioned between two gas pathways, as shown in Figure 3a and 3b. Additionally, the fluid pathway in contact with the PDMS membrane was extended by nearly 30% in length. The finite element simulation domains for the α – and β-chips are shown in Figure 3.

To experimentally measure the uptake of hydrogen gas into the β-chip, methanol was flowed into the fluid channel by means of a syringe pump located outside of the NMR spectrometer as shown in Figure 1. The chip was pressurized to 5 bar with hydrogen gas and its flow was controlled using a mass-flow controller set to 20 mL min^–1^. Dissolved hydrogen was detected by NMR in the 2.5 μL sample chamber on the chip.

Figure 3c shows the concentration of hydrogen in the sample chamber as a function of flow rate; 20 mM of sodium acetate was used as the concentration standard. The solid empty and black circles represent the NMR data for α- and β-chips, respectively. Error bars represent integrated rms noise in the spectra. Experimental NMR data for the α-chip was taken from ref (37). At 2 μL min^–1^ flow rate, the flowing liquid in both devices is fully saturated with hydrogen. However, as the flow rate increases to 10 μL min^–1^, the concentration of hydrogen in the β-chip is 11.3 mM versus only ∼6 mM in the α-chip. At a higher flow rate of 18 μL min^–1^ there is 3 times more hydrogen dissolved in the β-chip compared to the α-chip. The solid and dash-dotted lines are the FEM simulations and the gray shadows represent uncertainty due to fabrication tolerances of the chips. Simulations for both the α- and the β-chip are in good agreement with the experimental data for flow rates up to 10 μL min^–1^. Above this flow rate, the model consistently overestimates the hydrogen uptake. This discrepancy is not well understood yet, it was proposed that this could be due to the deformation of the PDMS membrane.^37^ However, simulations and experiments both suggest that the hydrogen uptake of the β-chip is higher by a factor of 2 for flow rates above 2 μL/min.

The PHIP performance of the microfluidic chip was compared to the results obtained by Eills et al. To this effect, the precursor solution containing 20 mM of propargyl acetate and 5 mM of rhodium catalyst flowed in the solution channel, while 5 bar of *para*-enriched hydrogen gas was supplied into the gas channel. The experimental setup is shown schematically in Figure 1. Hydrothermal sleeves were incorporated between the stripline detector and the microfluidic device housing, which facilitated efficient heating of the sample chamber up to 58 °C. The experiments and results are described in detail in the SI. Briefly, at the optimal flow rate of 5 μL min^–1^, the concentration of allyl acetate at 25 °C was determined to be 4.9 ± 0.2 mM, corresponding to a yield of 24.5 ± 1%. Compared to the results reported by Eills et al, this represents an increase in yield by a factor of 15. Increasing the temperature to 37 °C led to the concentration of allyl acetate of 7.0 ± 0.2 mM, corresponding to a yield of 35 ± 1%. This represents a further 10% increase in yield compared to the initial conditions. Elevation of the temperature to 47 °C led to a decrease in the concentration of allyl acetate to 5.4 ± 0.2 mM.

### Formation of ^13^C-Hyperpolarized Fumarate

The short lifetime of ^1^H polarization, of the order of seconds, limits application of ^1^H-hyperpolarization to track metabolic processes. This can be overcome by transferring the polarization to a longer-lived nucleus such as ^13^C or ^15^N. Hyperpolarized fumarate is a promising target for *in vivo* detection of necrosis and therefore has been extensively used as a hyperpolarization target.^43−46^ However, the trans-hydrogenation reaction to synthesize hyperpolarized fumarate is challenging as it is slow compared to the time frame in which the hyperpolarization returns to thermal equilibrium.^47^ As will be shown in the following, the enhanced hydrogen uptake of the β-chip together with the ability to run the reaction at slightly elevated temperature make it possible to hyperpolarize fumarate more efficiently.

**Figure 4:**
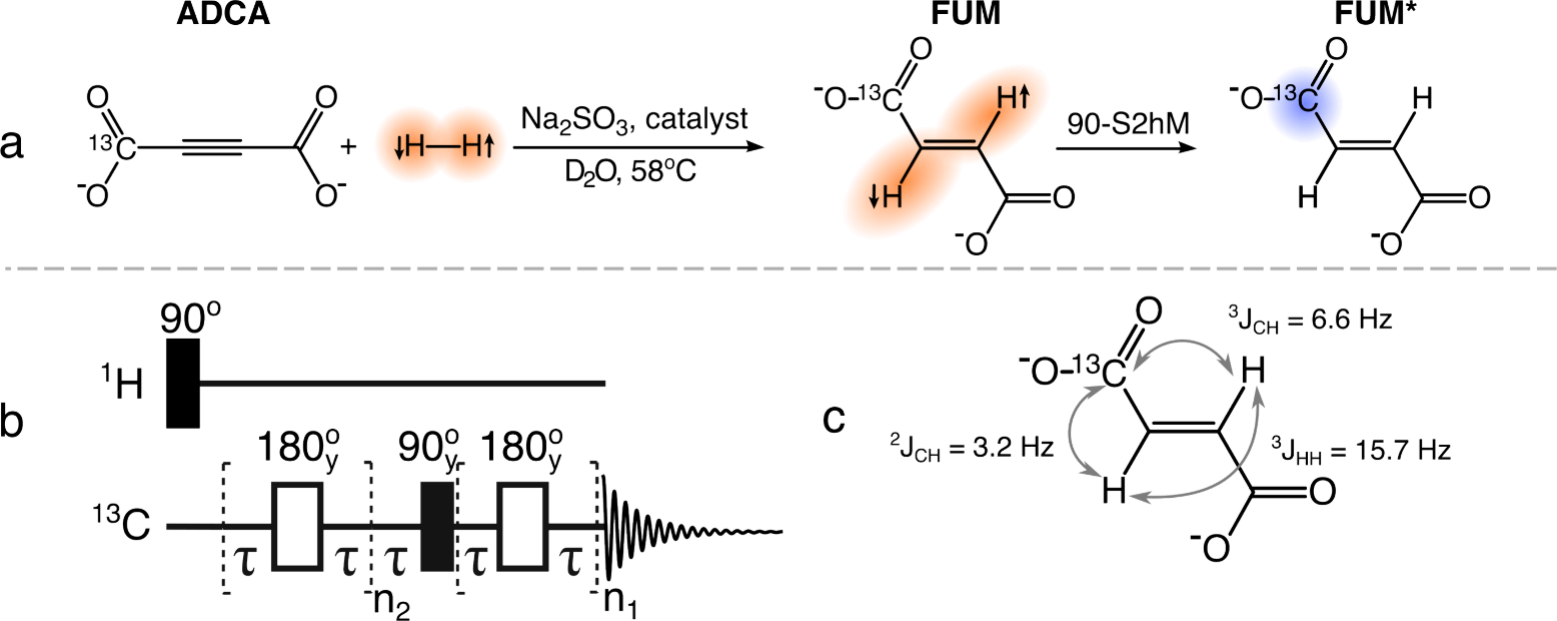
(a) Formation of ^13^C-hyperpolarized fumarate. Acetylene dicarboxylic acid [1-^13^C] disodium salt labeled as molecule **ADCA** reacts with parahydrogen in the presence of sodium sulfite and the catalyst  in D_2_O. The reaction results in a production of disodium [1-^13^C]fumarate, molecule **FUM**, with the two protons in a singlet state. Application of the S2hM pulse sequence converts the singlet state into observable ^13^C magnetization FUM*. (b) 90-S2hM pulse sequence used to transfer the polarization from the proton singlet state to carbon. (c) The *J*-coupling network of [1-^13^C]fumarate. The *J*-coupling values were taken from ref (43).

As shown in Figure 4a hyperpolarized fumarate was generated in aqueous solution via a reaction of [1 – ^13^C]-acetylenedicarboxylic acid disodium salt (**ADCA**) with *para* – hydrogen in the presence of a ruthenium catalyst, resulting in [1 – ^13^C]fumarate (**FUM**). Since the added protons are chemically and magnetically equivalent, a ^13^C label is required to to break the symmetry and enable observation of the spin order by NMR. The pulse sequence to convert the resulting singlet spin order into ^13^C magnetization is shown in Figure 4b. It consists of an initial purge pulse on the ^1^H channel, followed by an S2hM sequence^41^ on the ^13^C channel. This hydrogenation reaction is known to be affected by singlet–triplet (S-T) mixing, which can lead to a reduction of observable PHIP signal.^48^ S-T mixing occurs when molecules of hydrogen form intermediate hydride species with the catalyst metal center. At high magnetic fields the two protons experience a chemical shift difference in the hydride, which can lead to significant leakage from the proton singlet state (|*S*_0_⟩) to the central triplet state (|*T*_0_⟩).^49^ Partial signal cancellation occurs after S2M or S2hM sequences are applied which convert these states to either ^1^H or ^13^C magnetization but with opposite phases. There are methods for mitigating so-called S-T mixing.^48,50,51^ A π/2 ”purge” pulse prior to the S2M sequence was found to improve the efficiency of the sequence in microfluidic chips.^38^ The purge pulse removes the detrimental population of the |T_0_⟩ state by transferring it to the two outer |T_±_⟩ states where it has no effect on the polarization transfer. Here, the purge pulse was applied on the ^1^H channel prior to application of the S2hM sequence on the ^13^C channel as shown in Figure 4b.

**Figure 5:**
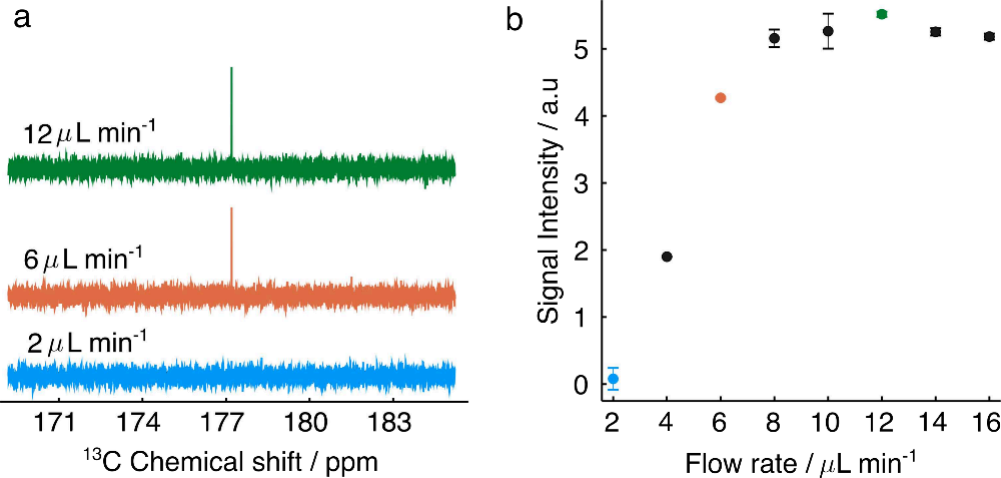
(a) ^13^C spectra of [1-^13^C]fumarate at different flow rates. (b) Hyperpolarized ^13^C signal intensity of [1-^13^C]fumarate as a function of fluid flow rate.

Figure 5a shows single scan ^13^C NMR spectra of ^13^C-hyperpolarized fumarate obtained at different flow rates using the setup depicted in Figure 1 and the β-chip at a temperature of 58 °C. At 2 μL min^–1^, the carbon signal is barely distinguishable from the noise but as the flow rate increases, the signal intensity increases. The change in signal intensity as a function of flow rate is displayed in Figure 5b. There is a gradual increase in signal intensity up to 8 μL min^–1^, followed by a plateau. This behavior is markedly different to what has been reported by Eills et al. for ^1^H hyperpolarization,^37^ which exhibited a sharp maximum at the optimum flow rate. At very low flow rates the time it takes for the product to be delivered into the sample chamber is greater that the spin relaxation time. This seems to be the case at 2 μL min^–1^ and below. It should be noted that since the polarization transfer only takes place in the sample detection region, it is the ^1^H singlet lifetime that is relevant here, not the ^13^C T_1_. Between 2 and 8 μL min^–1^ a gradually increasing amount of hyperpolarized material reaches the sample chamber. As shown in Figure 3, the hydrogen uptake decreases rapidly with increasing flow rate. It appears that this effect, which must lead to a decreasing yield of hydrogenation product with increasing flow rate, is almost perfectly compensated by the shorter amount of time needed for the product to reach the detection chamber at flow rates between 8 and 16 μL min^–1^. This gives rise to the hope that the ^13^C polarization could be substantially improved if the polarization transfer step could be carried out further upstream in the chip. Further experiments and detailed simulations are needed to clarify this point in support of a corresponding redesign of the microfluidic setup.

A straightforward way to quantify the enhancement factor is to run the same experiment with hydrogen in thermal equilibrium. Unfortunately, the concentration of fumarate was too low for the thermal ^13^C signal to be directly observed using our home-built transmission line probe, which is not optimized for sensitivity on the low frequency channel. To estimate the signal enhancement, the hyperpolarized spectrum was compared with a spectrum of 1 M d-Glucose-1-^13^C obtained after the application of  pulse (see SI). The SNR in the glucose spectrum is 2:1, while in the hyperpolarized spectrum of fumarate the SNR is 9:1. Since the glucose spectrum was obtained with 32 scans, the SNR from a single scan is . Accounting for the fact that glucose spectrum was obtained from a 1 M sample and the spectrum of fumarate was obtained from a 3 mM sample. This leads to the signal enhancement factor of , corresponding to 8.5% ^13^C polarization.

## Conclusions

In this work we have used finite element simulation results to inform the design of an optimized microfluidic device for performing PHIP reactions. FEM of the chip reported by Ostrowska et al.^39^ identified that inadequate uptake of hydrogen into the device is the limiting factor for the reaction, which resulted in submilimolar reaction yield. Introduction of an additional hydrogenation channel resulted in a 15-fold increase in the yield of hyperpolarized product compared with previously reported α-chip.^37^ Heating the sample chamber of the chip led to a further improvement of the yield. With these improvements, it has become possible for the first time to demonstrate the production and observation of the ^13^C hyperpolarized metabolite fumarate in a microfluidic device, with a ^13^C polarization of 8.5%. Further improvements are possible by optimization of the fluidic design, as well as by improvement of the ^13^C sensitivity of the microfluidic NMR probe. The present results represent an important step toward the integrated production of hyperpolarized materials and microfluidic cell culture.^15,16^ However, this requires integration of cleanup steps into the microfluidic system to remove the potentially toxic catalyst and reaction products. Research in this direction is underway in our laboratory, and will be reported at a later occasion.

## Data Availability

All raw experimental and simulation data have been deposited on zenodo.org, organized by figure.^52^

## Supporting Information Available

Data on allyl acetate *p*H_2_ experiments, details on the FEM simulation model, the ^1^H NMR spectrum of fumarate, as well as technical drawings of the microfluidic device (PDF)
