# Supplementary material for: Efficient Parahydrogen-Induced 13C Hyperpolarization on a Microfluidic Device
Source: J Am Chem Soc. 2024 Jun 25;146(27):18379–86. doi: 10.1021/jacs.4c03271 (PMC11240250; doi:10.1021/jacs.4c03271)
Supplement: Supplementary file 1 — ja4c03271_si_001.pdf [file ja4c03271_si_001.pdf]

Supporting Information: Efficient Parahydrogen Induced ^{13}C Hyperpolarization on a Microfluidic Device

Sylwia J. Barker,[†,‡] Laurynas Dagys,[†,¶] Malcolm H. Levitt,[†] and Marcel Utz[*,†,‡]

†*School of Chemistry, University of Southampton, Southampton, SO17 1BJ, United Kingdom*

‡*Institute of Microstructure Technology, Karlsruhe Institute of Technology, Karlsruhe, 76131, Germany*

¶*Institute of Chemical Physics, Vilnius University, Vilnius, 01513, Lithuania*

E-mail: marcel.utz@kit.edu

Contents

1 Multiphysics Modelling **2**

2 Characterization of Chip Performance Using Hyperpolarized Allyl Acetate **4**

2.1 Materials and Methods 5

2.2 Results and Discussion 6

3 ^{1}H NMR Spectra of $[1-^{13}C]$fumarate **11**

4 ^{13}C spectrum of 1M D−Glucose−1−^{13}C **11**

5 Raw Data **12**

6 β-chip Technical Drawings **12**

1 Multiphysics Modelling

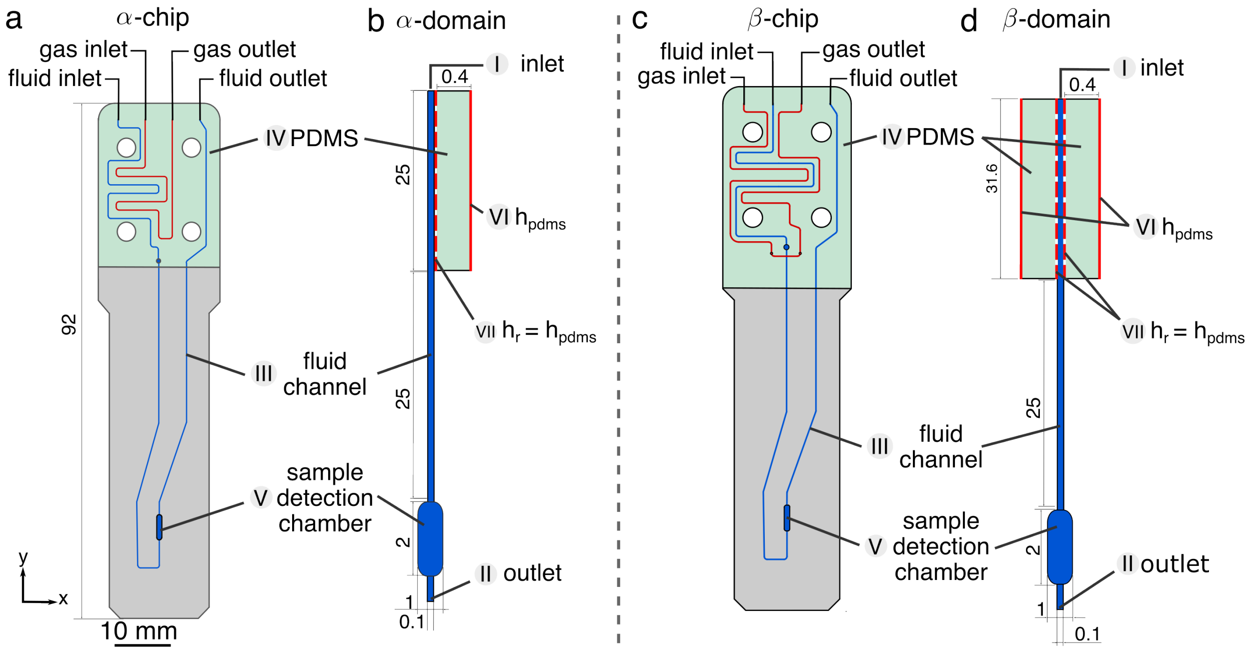

Figure S1 – a) The top view of the α-chip. b) Finite element simulation domain for the α-chip. Both adapted from Ref [1]. Available under CC BY 4.0. Copyright Ostrowska *et al.* c) The top view of the β-chip. d) Finite element simulation domain for the β-chip.

Finite element simulations were performed in COMSOL Multiphysics version 5.4. Fig. S1 a and b show the top view and the 2D simulation domain for the α-chip reported in Ref.[2] The simulation domain represents key functional parts of the α-chip which are: I) the inlet, II) the outlet, III) the fluid channel (50 mm length, 0.1 mm width) IV) the PDMS membrane (25 mm length, 0.4 mm width), and V) the sample detection chamber (2 mm length, 1 mm width). Although the gas channel has not been directly modeled, a constant concentration boundary VI (denoted as h_{pdms}) was enforced on the external boundary of the PDMS membrane. A steady-state of hydrogen concentration = 20 mM was imposed on the boundary. Hydrogen diffusion into the fluid channel

was facilitated by coupling the PDMS and fluid channel with a boundary condition VII $h_{pdms} = h_r$. The entrance thickness was set to $120 * 10^{-5}$ m, which resulted in a chip volume of 8.5 μL.

Fig. S1c depicts the top view of the β-chip where the fluid channel was interposed between two gas channels to increase the uptake of hydrogen into the chip. To simulate this, additional PDMS membrane, h_{pdms} and h_r boundaries were added in the β-simulation domain as shown in Fig. S1d. Furthermore, the length of the fluid pathway in contact with the PDMS membrane and the membrane itself were elongated by 6.6 mm. The total volume of the β-chip was calculated as 7 μL.

The Laminar Flow module in COMSOL was used to find the flow pattern in the fluid channel. This was done by solving the Navier-Stokes equations for laminar flow regime for incompressible fluid:

$$\nabla \cdot \mathbf{u} = 0, \tag{1}$$

where $\nabla \cdot$ is the divergence and $\mathbf{u}$ is the flow velocity. The conservation of momentum is given by:

$$\rho \frac{\partial \mathbf{u}}{\partial t} + \rho \mathbf{u} \cdot \nabla \mathbf{u} = -\nabla p + \eta \nabla^2 \mathbf{u}, \tag{2}$$

where the changes in momentum ($\rho \frac{\partial \mathbf{u}}{\partial t}$) are caused by the net momentum convected out of the control volume by the fluid flow ($\rho \mathbf{u} \cdot \nabla \mathbf{u}$), the surface ($\nabla p$), and the body forces $\eta \nabla^2 \mathbf{u}$.

Table 1 – Parameters used in COMSOL simulations for hydrogen uptake in the $\alpha-$ and $\beta-$domains. **PR** refers to propargyl acetate, **AL** represents allyl acetate.

**Simulation Parameters** / units	
Flow Rate / μL min^{-1}	2...50
Diffusion Coefficient / m^2 s^{-1}	$1 \cdot 10^{-9}$
[Hydorogen]$_0$ / mol m^{-3}	20
[**PR**]$_0$,[Rh(dppb)BF_4]$_0$,[**AL**]$_0$/ mol m^{-3}	0
Temperature / K	298.15

The Transport of Dilute Species module was used to simulate the uptake of hydrogen into the chips. The convection-diffusion equation for hydrogen becomes:

$$\frac{\partial c_{H_2}}{\partial t} = D\nabla^2 c_{H_2} - \mathbf{u} \cdot \nabla c_{H_2}, \tag{3}$$

All simulation parameters are listed in Table 1.

2 Characterization of Chip Performance Using Hyperpolarized Allyl Acetate

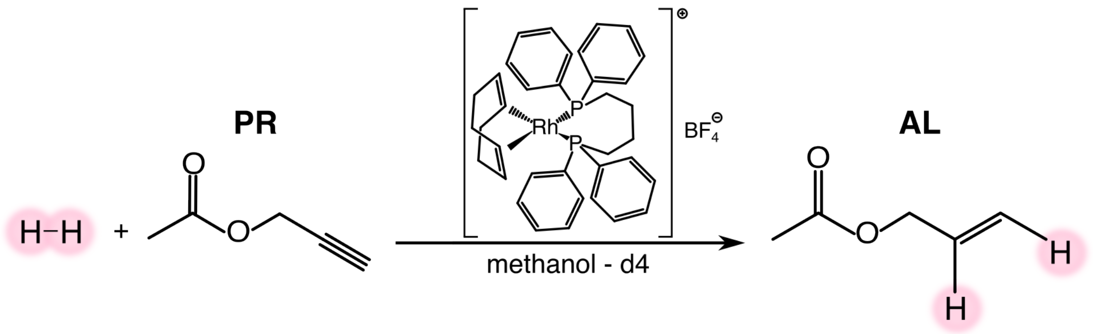

Figure S2 – Propargyl acetate hydrogenation reaction scheme. Propargyl acetate **PR** reacts with parahydrogen in the presence of an organometallic catalyst to produce hyperpolarized allyl acetate **AL**.

Alkyne esters represent an important class of targets as they offer the possibly to hyperpolarize a range of carboxylate containing molecules such as pyruvate or lactate via the side-arm hydrogenation (PHIP-SAH) strategy.[3–6] Eills et al have demonstrated production of hyperpolarized allyl acetate (**AL**) using the microfluidic device shown in Fig. S1 a.[7] This chemical transformation entails the utilization of parahydrogen gas and a rhodium catalyst to catalyze the hydrogenation of propargyl acetate (**PR**). As reported in the main article, we have used the same reaction as a benchmark to be able to compare the performance of the original (α-chip) and the improved (β-chip) designs.

2.1 Materials and Methods

The precursor solution contained 5 mM of [1,4-bis(diphenylphospino)butane](1,5-cycloctadiene) rhodium (I) tetrafluoroborate and 20 mM of propargyl acetate dissolved in methanol-d_4. Parahydrogen gas pressure was set to 5 bar. The nutation frequency for RF pulses was 125 kHz for protons. Spectra were collected with a 16 ppm spectral width and 8 k data points. Data was acquired continuously while varying the flow rate from 2 to 12 $\mu L\,min^{-1}$ in steps of 1 $\mu L\,min^{-1}$ and from 14 to 20 $\mu L\,min^{-1}$ in steps of 2 $\mu L\,min^{-1}$. At each flow rate, 20 single scan spectra were acquired after the application of a $\frac{\pi}{4}$ pulse with a recycle delay of 30 s. At these experimental conditions, it took $\sim$ 2 minutes for the hyperpolarized signal to reach the sample chamber and stabilise therefore the first 10 transients at each flow rate were discarded to ensure that only steady-state data was taken into consideration.

Spectral integrals from PASADENA experiments were obtained by fitting two Lorentzian functions to the H^h signal. This process is illustrated in Fig. S3, where the experimental data is represented by the blue line and the fit is shown as the black solid line. Only the positive lobe of the antiphase peak was integrated, as indicated by the shaded region.

The fitting routine was performed using Julia 1.6[8] with the NMR package written by Marcel Utz.[9]

Reference spectra were obtained using hydrogen in thermal equilibrium instead of parahydrogen. 10 mM of isopropanol (IPA) was added as the concentration standard while other conditions remained identical. Addition a minimal amount of another solvent is not expected to negatively impact the reaction.

To obtain reference spectra, the precursor solution was flowed at 5, 7 and 9 $\mu L\,min^{-1}$ for 25°C, 37°C, and 47°C, respectively. The pressure of hydrogen in thermal equilibrium was set to 5 bar, all other conditions remained identical. Each reference spectrum is an average of 512 transients with a 30 s recycle delay. The yield of allyl acetate was found by comparing the integral of signal H^f peak at 5.9 ppm to the intensity of the isopropanol peak at 1.1 ppm and accounting for the difference

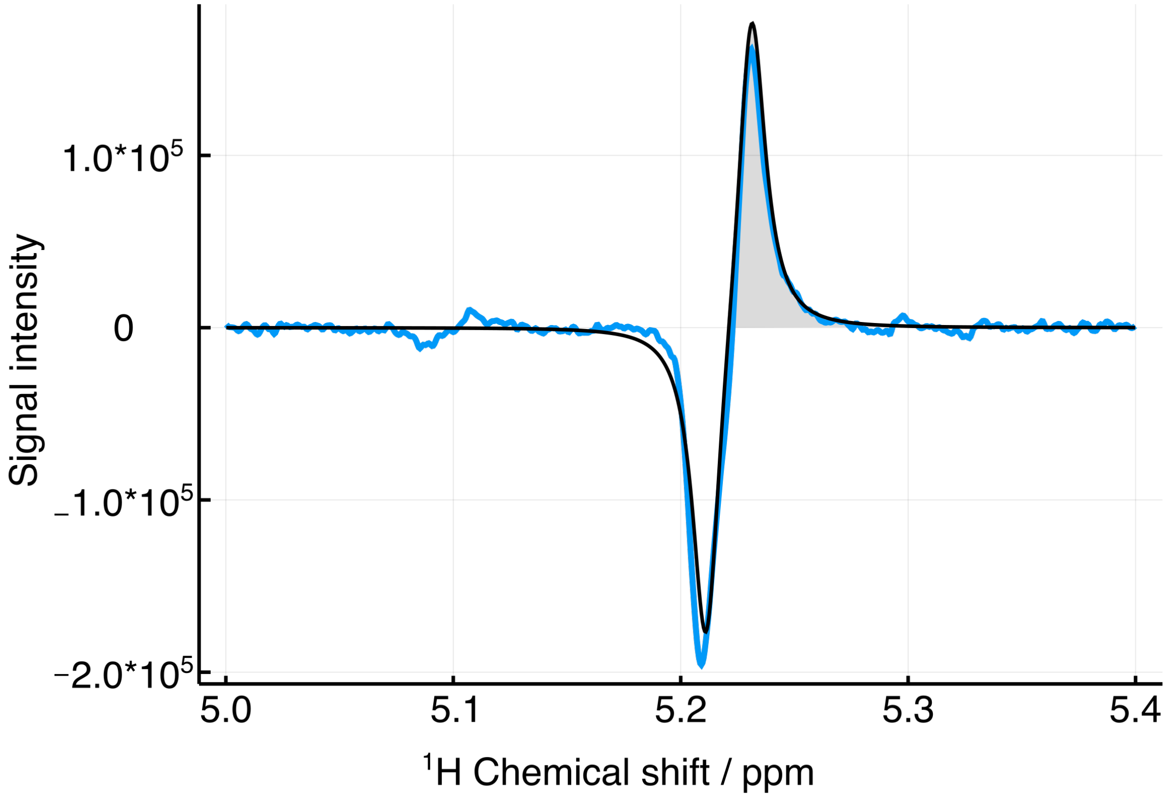

Figure S3 – The fitting routine used to derive integral values from PASADENA experiments. The hyperpolarized spectrum of allyl acetate is presented in blue, while the fitted Lorentzian function is shown in black. The integral was obtained by calculating the area under the positive lobe of the antiphase peak, which is highlighted as the grey shaded region.

in the number of protons. The yield of propyl acetate was calculated as described above, from the integral of H^i peak at 4.33 ppm to the intensity of the isopropanol peak. The enhancement factor was obtained by calculating the SNR in the thermal and reference spectra and accounting for the difference in the number of scans.

2.2 Results and Discussion

The PHIP reaction was performed by flowing the precursor solution containing 20 mM of propargyl acetate **PR** and 5 mM of rhodium catalyst into the solution channel and delivering 5 bar of *para*-enriched hydrogen gas into the gas channel. Fig. S4 a shows a single scan proton NMR spectrum acquired after a $\frac{\pi}{4}$ pulse. The spectra were acquired during a steady-state experiment, with

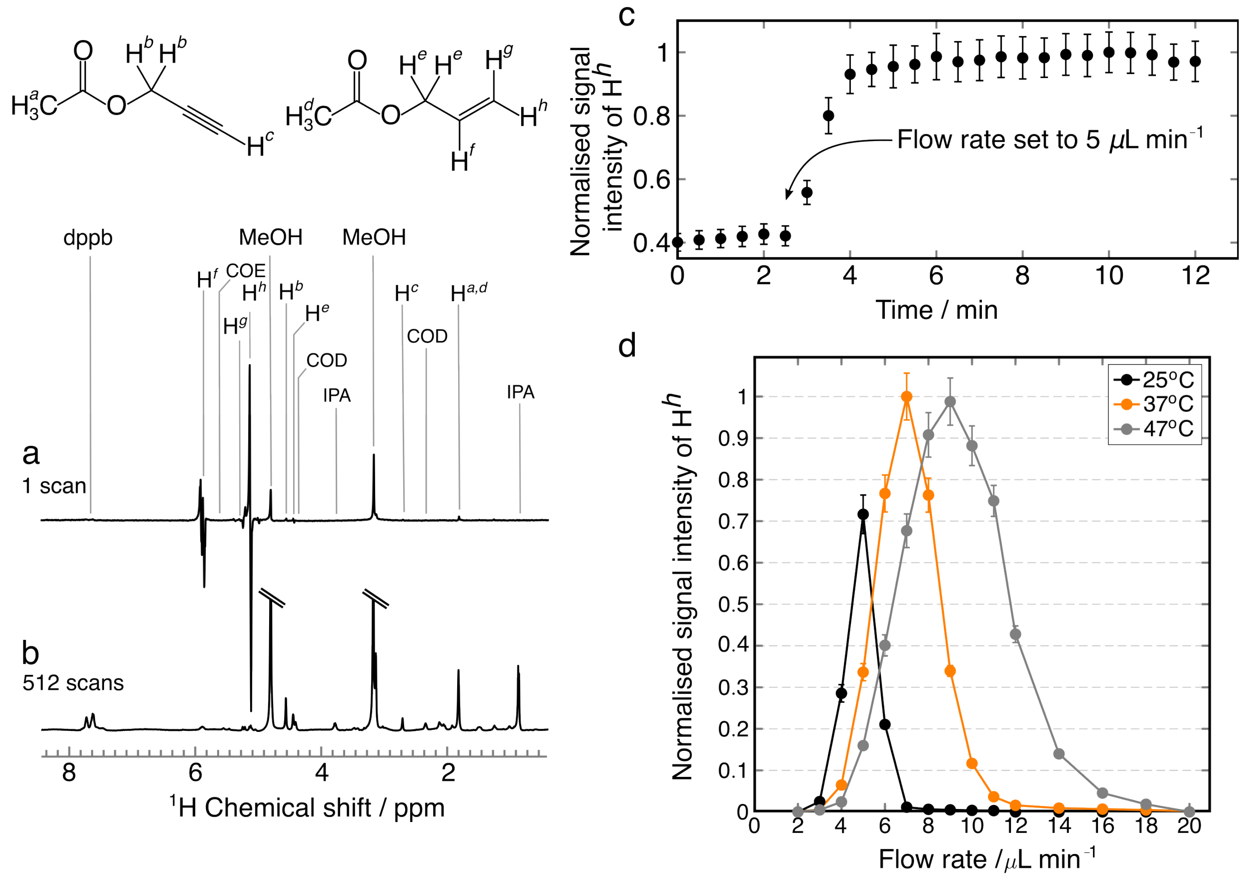

Figure S4 – a) Single-scan proton spectrum obtained with *para*-hydrogen. b) Reference spectrum obtained with hydrogen in thermal equilibrium. Methanol peaks have been suppressed for clarity. c) Build-up of hyperpolarized signal (H^h) after changing the flow rate from 4 to 5 $\mu L\,min^{-1}$. d) Flow-rate dependence of the hyperpolarized allyl acetate yield at three different temperatures. Black, orange and gray dots represent the experimental data for data obtained at 25°C, 37°C and 47°C respectively. Solid lines have been included as guides to the eye.

the solution continuously flowed at a rate of 5 μL min^{-1}. The hyperpolarized spectrum contains an antiphase doublet at 5.2 ppm corresponding to protons H^h and an antiphase multiplet at 5.9 ppm corresponding to protons H^f. This is compared with a 512-scans reference spectrum obtained with hydrogen in thermal equilibrium shown in Fig. S4b. Each scan was acquired after a $\frac{\pi}{2}$ pulse with a recycle delay of 30 s. From the ratio of the signal intensity in the reference and hyperpolarized spectra, the ^{1}H polarization was estimated. In the reference spectrum, the SNR was found to be 9:1, while it was 300:1 in the hyperpolarized spectrum. Since the reference spectrum was obtained using 512 scans, the SNR resulting from a single scan would be $9/\sqrt{512} \approx 0.40$. Therefore, the signal enhancement factor is $\varepsilon \approx 300/0.4 \approx 750$. At the field of 11.7 T and temperature of 25°C this corresponds to 3% ^{1}H polarization.

At 5 μL min^{-1} flow rate, the concentration of propargyl acetate was 4.9 ± 0.2 mM, which corresponds to 24.5 ± 0.25% yield. This was calculated by comparing the intensity of the isopropanol peak at 1.14 ppm in the reference spectrum to the intensity of the H^f peak and accounting for the difference in the number of protons. Therefore by introducing an additional gas channel the yield of allyl acetate was increased over 15-fold compared with the α-chip.[7]

Fig. S4c shows the buildup of a hyperpolarized signal H^h after changing the flow rate from 4 to 5 μL min^{-1}. NMR spectra were acquired every 30 s using a $\frac{\pi}{4}$ excitation pulse. The signal intensity begins to rise 30 s after the target flow rate was set to 5 μL min^{-1} and it reaches a steady-state after 2 minutes. Similar results were shown in Ref[7] where the signal intensity also took just 2 minutes to build-up. After reaching a steady-state the system provides a remarkable stability of the signal.

Fig. S4d shows the normalised signal intensity of proton (H^h) as a function of flow rate at three temperatures. The black, orange and gray solid circles correspond to experimental data obtained at 25°C, 37°C and 47°C respectively. Each data point on the graph is a mean of 10 integrals of peak H^h obtained from consecutive acquisitions. As the temperature is increased form 25°C to 37°C, the maximum of the signal increases by 30%. Upon further 10°C temperature increase, the maximum signal decreases by 2%. Increasing the temperature by 10°C shifts the position of the

maximum by +2 μL min^{-1}. At 25°C the maximum is located at 5 μL min^{-1}, while at 47°C it is at 9 μL min^{-1}. The position of the maximum represents a balance between the rate of relaxation of the hyperpolarized product and the hydrogen uptake into the chip. At low flow rates such as 2 μL min^{-1}, the residence time of the hyperpolarized product in the fluid channel is very long therefore it fully relaxes before it reaches the sample chamber. As the flow rate increases, the product is delivered faster to the sample chamber and less of it is lost due to relaxation hence the steep spike from 2 μL min^{-1} till the maximum. As the temperature increases so do the reaction rates hence the position of the maximum shifts to a higher flow rate as the product needs to be delivered faster into the sample chamber.

In order to determine the yield of allyl acetate at the optimum flow rate, the experiment was repeated using hydrogen in thermal equilibrium. 10 mM isopropanol was used as the concentration standard and the thermal spectra are shown in Fig. S5. The results are listed in Table 2.

The concentration of allyl acetate at 25°C was found to be 4.9 ± 0.2 mM, which corresponds to 24.5 ± 1% yield. At 37°C the concentration was found to be 7.0 ± 0.2 mM, resulting in the yield of 35.0 ± 1.0%. This is a further increase of 10% in the yield. Increasing the temperature further to 47°C, resulted in a drop in the concentration of **AL** to 5.4 ± 0.2 mM. At 37°C the reaction is most efficient and even leads to the formation of propyl acetate, as evidence by a H^i peak at 4.33 ppm in Fig. S5, which is absent at other temperatures. The concentration of propyl acetate was calculated as 1.2 ± 0.2 mM.

The enhancement factor for reactions conducted at 37°C and 47°C was found to be 1060 and 930, indicating proton polarizations of 4.1% and 3.5%, respectively. The polarization level for the α-chip was reported as 8%,[7] which is a much higher polarization than obtained in the β-chip.

Table 2 – Experimental results for the PHIP reaction in the β-chip performed at 25°C, 37°C and 47°C.

**T / °C**	**q / μL min^{-1}**	**[AL] / mM**	ε	^{1}H **polarization / %**	**Molar polarization / μ M**
25	5	4.9 ± 0.2	750	3.0	147 ± 6
37	7	7.0 ± 0.2	1060	4.1	287 ± 8
47	9	5.2 ± 0.2	930	3.5	182 ± 7

However, for *in situ* studies spin polarization may not be a sufficient measure as the total number of spins detected also depends on the molar concentration of the species. Instead, molar polarization should be considered, which is defined as the product of the polarization level and the concentration of the species.[10] Molar polarization for the β-chip was calculated as 147 $\pm$ 6, 287 $\pm$ 8, 182 $\pm$ 7 μM, for reactions preformed at 25°C, 37°C, and 47°C, respectively, while the molar polarization for the α-chip was less than 40 μM. By implementing an additional hydrogenation pathway in the β-chip the molar polarization has nearly quadrupled compared with the α-chip. Elevating temperature of the reaction by 10°C further approximately doubled the molar polarization. However at 47°C, molar polarization decreased.

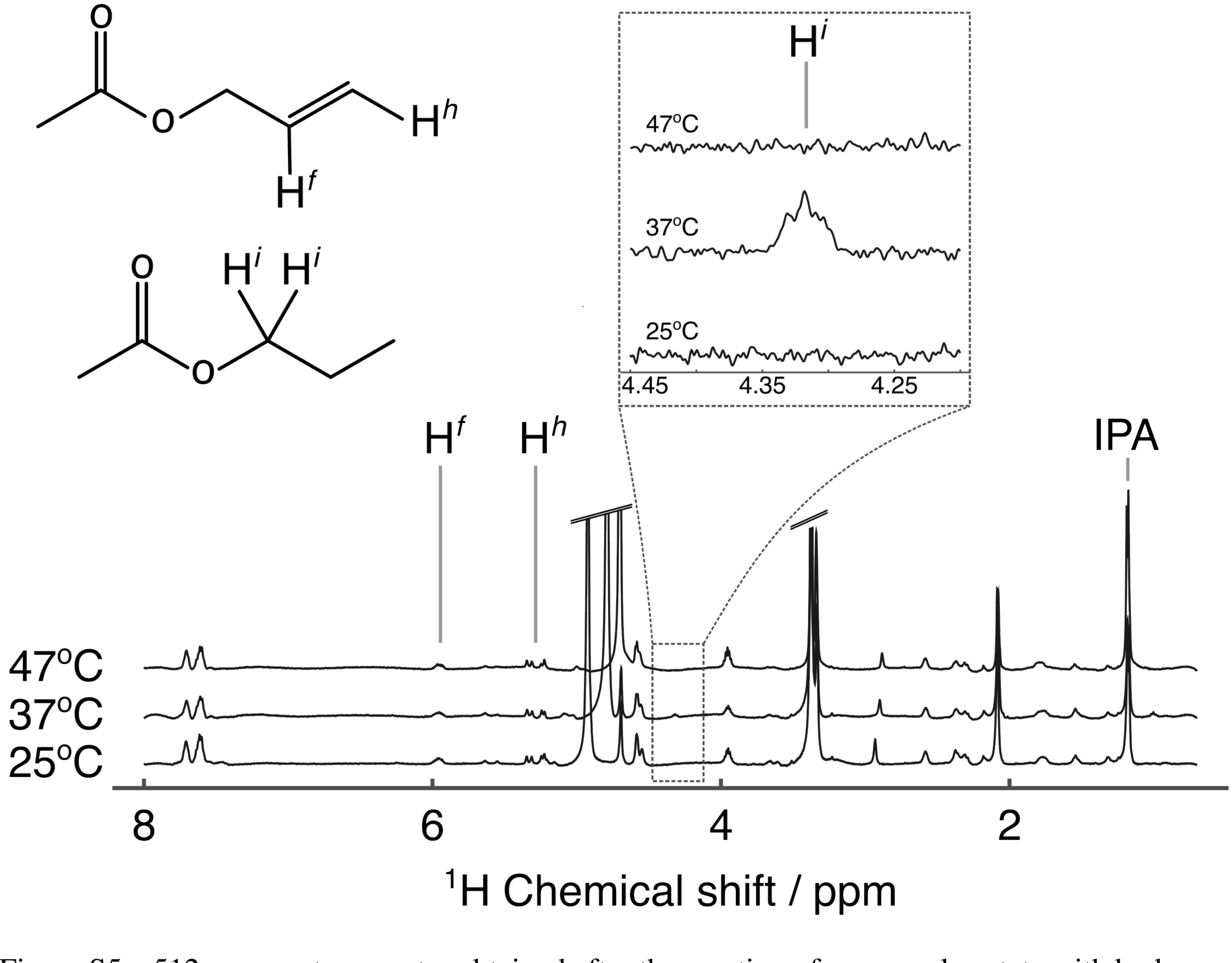

Figure S5 – 512 scan proton spectra obtained after the reaction of propargyl acetate with hydrogen in thermal equilibrium at 25°C, 37°C, and 47°C. Spectra were obtained at steady-state flow rate of 5, 7, and 9 μL min^{-1}, respectively. The key species used in the yield calculations are highlighted. 10x magnification of the 4.25 - 4.45 ppm region reveals the propyl acetate peak H^i.

3 ^{1}H NMR Spectra of $[1-^{13}C]$fumarate

Fig. S6 shows the thermal spectrum of the precursor solution flowed at 12 μL min^{-1} used to produced fumarate. The spectrum is an average of 256 scans. Fumarate yield was calculated by comparing the integral of the H^a peak at 6.8 ppm to the catalyst Cp* peak at 2.35 ppm and accounting for the difference in the number of protons.

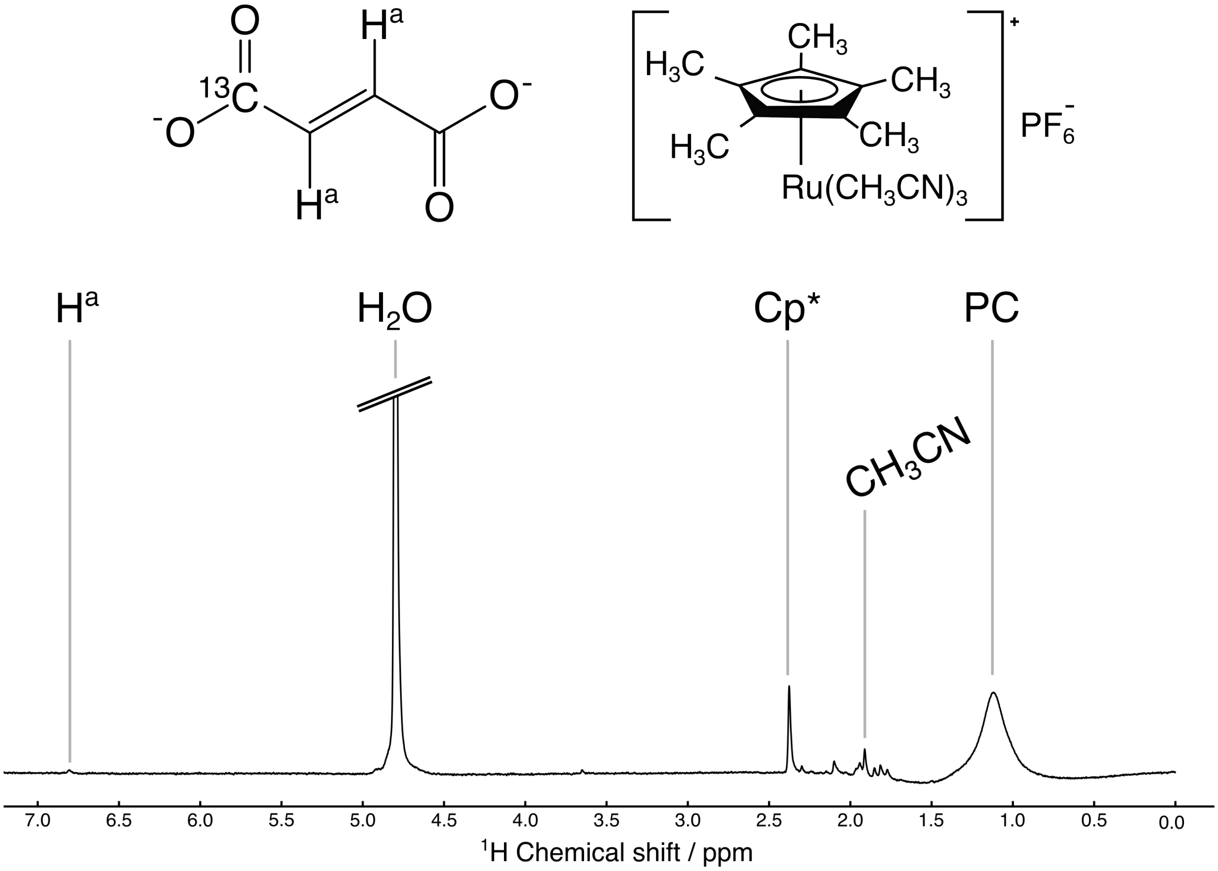

Figure S6 – ^{1}H spectrum of the precursor solution to produce $[1-^{13}C]$fumarate.

4 ^{13}C spectrum of 1M D$-$Glucose$-1-^{13}$C

To obtain a ^{13}C reference spectrum, the β-chip was filled with a solution of 1M D-Glucose-1-^{13}C dissolved in D_2O. The probe delivered nutation frequencies for ^{13}C RF pulses of 12.5 kHz. Spectra

were collected with a 200 ppm spectral width, and 8 k data points were acquired and were averaged over 32 scans.

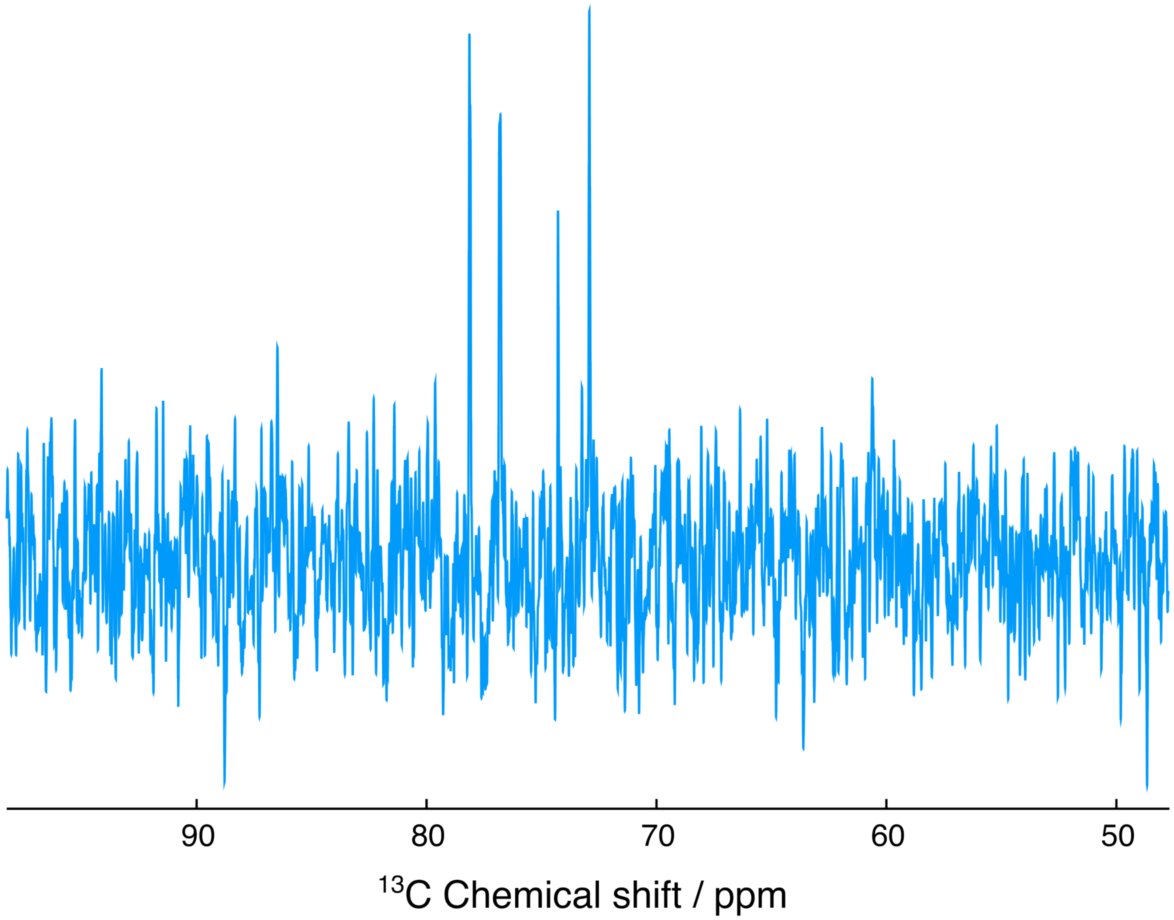

Figure S7 – ^{13}C spectrum of 1M D-Glucose-1-^{13}C averaged over 32 scans.

5 Raw Data

The raw data for this work, organised by figure, is available on Zenodo.org[11]

6 β-chip Technical Drawings

Fig. S 8 shows the technical drawings of the β-chip in 1:1 scale.

References

(1) Ostrowska, S. J.; Rana, A.; Utz, M. Spatially Resolved Kinetic Model of Parahydrogen Induced Polarisation (PHIP) in a Microfluidic Chip. *ChemPhysChem* **2021**, *22*, 2004–2013.

(2) Eills, J.; Hale, W.; Sharma, M.; Rossetto, M.; Levitt, M. H.; Utz, M. High-Resolution Nuclear Magnetic Resonance Spectroscopy with Picomole Sensitivity by Hyperpolarization on a Chip. *J. Am. Chem. Soc.* **2019**, *141*, 9955–9963.

(3) Stevanato, G.; Ding, Y.; Mamone, S.; Jagtap, A. P.; Korchak, S.; Glöggler, S. Real-Time Pyruvate Chemical Conversion Monitoring Enabled by PHIP. *J. Am. Chem. Soc.* **2023**, *145*, 5864.

(4) Carrera, C.; Cavallari, E.; Digilio, G.; Bondar, O.; Aime, S.; Reineri, F. ParaHydrogen Polarized Ethyl-[1-^{13}C]pyruvate in Water, a Key Substrate for Fostering the PHIP-SAH Approach to Metabolic Imaging. *ChemPhysChem* **2021**, *22*, 1042–1048.

(5) Reineri, F.; Boi, T.; Aime, S. ParaHydrogen Induced Polarization of ^{13}C Carboxylate Resonance in Acetate and Pyruvate. *Nat. Commun.* **2015**, *6*, 1–6.

(6) Cavallari, E.; Carrera, C.; Aime, S.; Reineri, F. Studies to Enhance the Hyperpolarization Level in PHIP-SAH-produced C13-Pyruvate. *J. Magn. Reson.* **2018**, *289*, 12–17.

(7) Eills, J.; Hale, W.; Sharma, M.; Rossetto, M.; Levitt, M. H.; Utz, M. High-Resolution Nuclear Magnetic Resonance Spectroscopy with Picomole Sensitivity by Hyperpolarization on a Chip. *J. Am. Chem. Soc.* **2019**, *141*, 9955–9963.

(8) Jeff Bezanson, S. K. The Julia Programming Language. 2022; `https://www.julialang.org`, [Online; Accessed 04 Mar 2024].

(9) Utz, M. NMR Package for Julia Programming Language. `https://github.com/marcel-utz/NMR.jl.git`, 2021; [Online; Accessed 04 Mar 2024].

(10) Knecht, S. et al. Rapid Hyperpolarization and Purification of the Metabolite fumarate in Aqueous Solution. *Proc. Natl. Acad. Sci. U.S.A.* **2021**, *118*, e2025383118.

(11) Ostrowska, S.; Dagys, L.; Utz, M.; Levitt, M. Raw Data for: Efficient Parahydrogen Induced 13C Hyperpolarization on a Microfluidic Device. `https://zenodo.org/records/11108345`.

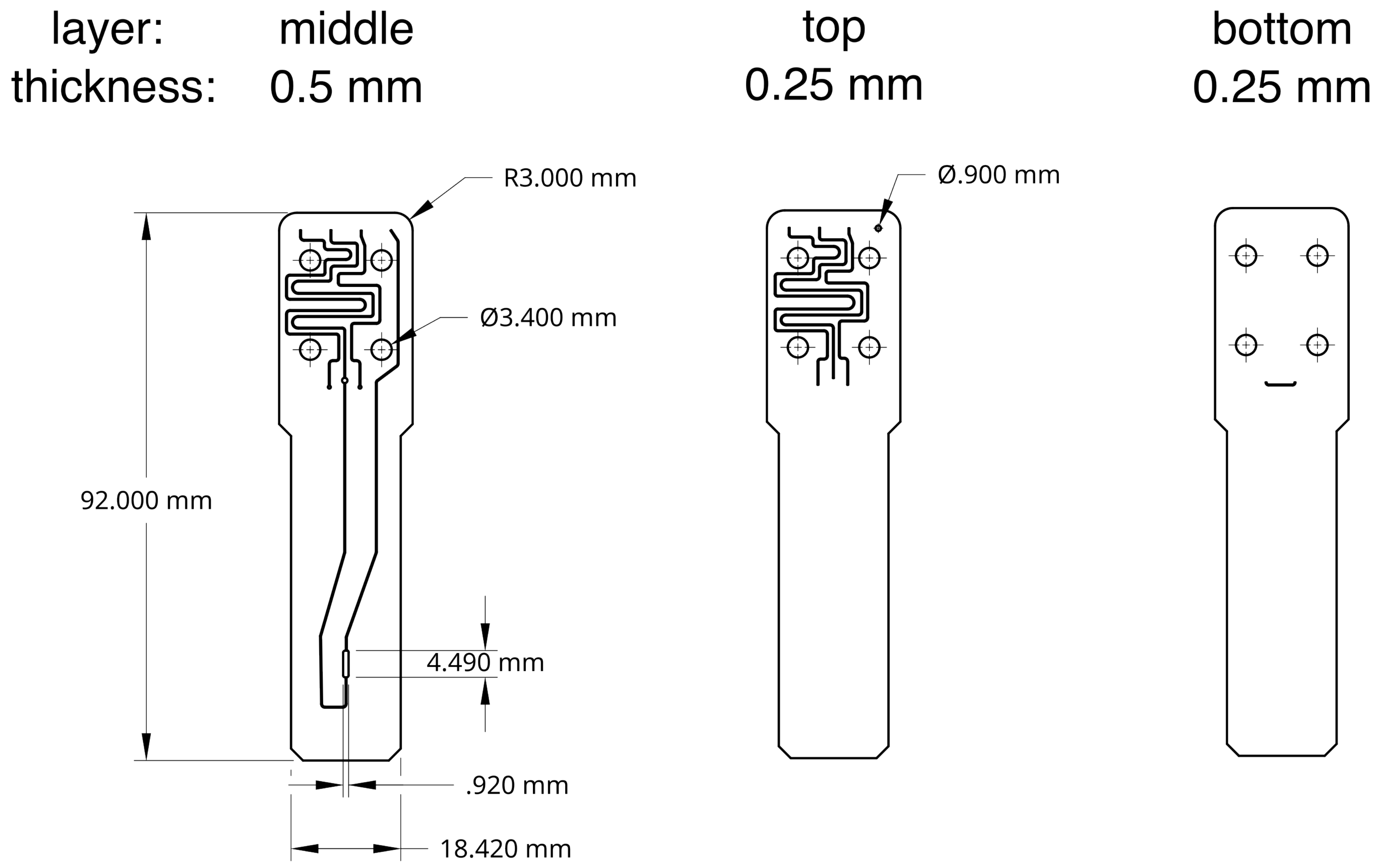

Figure S8 – Technical drawings of the β-chip. Scale 1:1
